# The Mucosal Innate Immune Response to *Cryptosporidium parvum*, a Global One Health Issue

**DOI:** 10.3389/fcimb.2021.689401

**Published:** 2021-05-25

**Authors:** Charles K. Crawford, Amir Kol

**Affiliations:** Department of Pathology, Microbiology, & Immunology, School of Veterinary Medicine, University of California, Davis, Davis, CA, United States

**Keywords:** *Cryptosporidium parvum*, innate immune response, One Health, intestinal parasite, intestinal epithelium

## Abstract

*Cryptosporidium parvum* is an apicomplexan parasite that infects the intestinal epithelium of humans and livestock animals worldwide. Cryptosporidiosis is a leading cause of diarrheal-related deaths in young children and a major cause of economic loss in cattle operations. The disease is especially dangerous to infants and immunocompromised individuals, for which there is no effective treatment or vaccination. As human-to-human, animal-to-animal and animal-to-human transmission play a role in cryptosporidiosis disease ecology, a holistic ‘One Health’ approach is required for disease control. Upon infection, the host’s innate immune response restricts parasite growth and initiates the adaptive immune response, which is necessary for parasite clearance and recovery. The innate immune response involves a complex communicative interplay between epithelial and specialized innate immune cells. Traditional models have been used to study innate immune responses to *C. parvum* but cannot fully recapitulate natural host-pathogen interactions. Recent shifts to human and bovine organoid cultures are enabling deeper understanding of host-specific innate immunity response to infection. This review examines recent advances and highlights research gaps in our understanding of the host-specific innate immune response to *C. parvum*. Furthermore, we discuss evolving research models used in the field and potential developments on the horizon.

## Introduction

*Cryptosporidium parvum* is an apicomplexan parasite that causes potentially life-threatening infectious diarrhea in infants and neonate calves with no available FDA-approved vaccine (Abrahamsen et al., 2004; Abubakar et al., 2007). Nitazoxanide is approved for use in adult, immunocompetent patients, but is not effective or approved for use in the most vulnerable populations: infants and immunocompromised patients (Abubakar et al., 2007). Infected hosts (humans and cows) shed billions of highly infectious and environmentally stable parasites (Nydam et al., 2001; Zambriski et al., 2013). The parasite is transmitted zoonotically and between humans worldwide (Bouzid et al., 2013; Ryan et al., 2016; Hatam-Nahavandi et al., 2019), and it contaminates drinking water sources (Chique et al., 2020), recreational swimming sites (Li et al., 2019), soils (Nag et al., 2020), and aquaculture environments (Marquis et al., 2015). Wildlife is further impacted by cryptosporidiosis (Ziegler et al., 2007). As such, a comprehensive and interdisciplinary research approach is required to eliminate this significant source of global disease burden. Cryptosporidiosis is one of three etiologies responsible for the most global diarrheal deaths in children younger than five years of age (Checkley et al., 1997; Ong et al., 2005; Gormley et al., 2011; Kotloff et al., 2013; Khalil et al., 2018). In the U.S. *C. parvum* caused the largest waterborne pathogen outbreak in American history (Corso et al., 2003), and 444 outbreaks of cryptosporidiosis were reported from 2009-2017, leading to an estimated 750,000 individual cases per year (Scallan et al., 2011; Gharpure et al., 2019). *Cryptosporidium* has even been included as a relevant biological threat agent by the CDC (Rotz et al., 2002).

While the role of CD4^+^ T cells in clearing infection is well studied, the role of the innate immune response within the parasite’s natural hosts (i.e. human and cattle) is not fully understood. We will succinctly review current advances in our understanding of the mucosal innate immune response to *C. parvum*, and innovative models that have the potential to elucidate such responses within clinically relevant hosts. We will highlight key knowledge gaps and future research opportunities.

## Innate Immune Response

The intestinal innate immune system, comprised of the gut epithelium and specialized innate immune cells, is the first line of defense against *C. parvum* infection. Innate immunity restricts the expansion and growth of the parasite and initiates the adaptive response. Understanding the innate immune response to *C. parvum* infection in its native hosts is critical in building a ‘One Health’ strategy to limit *Cryptosporidium’s* devastating impact on global health, agriculture, and the environment (Ziegler et al., 2007; Laurent and Lacroix-Lamande, 2017; Ivanova et al., 2019).

### Intestinal Epithelial Cells 

Intestinal epithelial cells that line the gut epithelium create a physical barrier between luminal content and internal tissues.

Because *C. parvum* infects intestinal epithelial cells and does not invade deeper tissues, the epithelium is particularly important regarding the immune response to *C. parvum*.

Primary bovine intestinal epithelial cell infection by *C. parvum* leads to activation of the inflammatory transcription factor NF-κB; this increases expression of the long noncoding RNA NR_045064 (Li et al., 2018) and induces the transcription of numerous inflammatory mediators, primarily CXCL8 (aka IL-8) and TNFα, a response primarily mediated by Toll-like receptor-2 (TLR2) and TLR4 (Yang et al., 2015). *C. parvum* infection induces an increase in TLR4 expression, regulated by suppression of the noncoding miRNA, *let-7i* (Chen et al., 2007). TLR2 and TLR4 activation by *C. parvum* and subsequent NF-κB nuclear translocation induces the release of antimicrobial peptides LL-37 and β-defensin-2 (Chen et al., 2005). Despite this, TLR2 and TLR4 deficiency did not increase parasite load in neonatal mice; however, direct comparisons are difficult given the different models and experimental designs (Lantier et al., 2014).

Evidence suggests that intracellular recognition of *C. parvum* via NOD-like receptors (NLR) and subsequent activation of the inflammasome complex is an important innate response to infection. IL-18, a product of the inflammasome complex, is elevated in human epithelial cell lines following *C.* parvum infection (McDonald et al., 2006); moreover, IL-18 knockout and inflammasome components caspase-1 or ASC knockout mice are more susceptible to *Cryptosporidium* infection than control mice (Ehigiator et al., 2005; McNair et al., 2018; Sateriale et al., 2021). IL-1β, the second key product of inflammasome activation, was not increased post-infection, nor was there an effect on infection susceptibility in IL-1β knockout mice (McNair et al., 2018). The latter findings are corroborated by the fact that parasite shedding was strongly increased in mice lacking NLRP6, which induces IL-18 secretion, but not in mice lacking other inflammasome-forming NLRs including NLRP3, NLRP1b, Aim2, and NLRc4 that primarily induce IL-1β secretion (Sateriale et al., 2021).

Antimicrobial peptides include small positively-charged polypeptides that elicit antimicrobial effects against a variety of pathogens including bacteria, fungi, viruses, and protozoan parasites (Mahlapuu et al., 2016). Phospholipases (Carryn et al., 2012) and the antimicrobial peptides β-defensin-1, β-defensin-2, and LL-37 can kill *C. parvum* (Giacometti et al., 1999). Part of the TLR signal response by epithelial cells includes the release of LL-37 and β-defensin-2, and these antimicrobial peptides bind to free *C. parvum* to directly enact their effects (Chen et al., 2005). LL-37 and α-defensin-2 are increased in response to the rise in the inflammasome product, IL-18, in human cell lines (McDonald et al., 2006). However, *C. parvum* influences epithelial cells by inhibiting the production of other antimicrobial peptides including β-defensin-1 by an undiscovered mechanism (Zaalouk et al., 2004), and CCL20 by a *C. parvum*-induced rise in miR21 (Guesdon et al., 2015).

*C. parvum* infection is restricted to a parasitophorous vacuole on the apical side of the intestinal epithelium, therefore chemokine and cytokine release from infected epithelial cells is critical in the recruitment of specialized immune cells that facilitate parasite clearance (Laurent et al., 1999). Activation of TLRs by *C. parvum* induces the NF-κB signaling pathway causing the basolateral release of Growth Regulated Oncogene-α (GRO-α) (Yang et al., 2015) and CXCL8, which are key neutrophil chemoattractant molecules (Laurent et al., 1997). Additionally, in the neonatal mouse model, several chemokines including CCL2, CCL5, CXCL10, and CXCL9 are released, which recruit various immune cells to the infection site (Lacroix-Lamande et al., 2002; Auray et al., 2007; Lantier et al., 2013). Chemokine-induced immune cell recruitment is critical in the response to *C. parvum*, as evidenced by the increased susceptibility to infection of mice deficient in chemokine receptors, even in spite of redundancy in immune cell recruitment processes (Lacroix-Lamande et al., 2008; Lantier et al., 2013).

Another defense against intracellular pathogens is apoptosis of the host cell, and infection by *C. parvum* initiates apoptosis of infected and surrounding epithelial cells through Fas and Fas-L interactions (Chen et al., 1999). However, within hours post-infection, *C. parvum* in one life stage, the trophozoite, inhibits apoptosis, likely to facilitate growth within the host cell, by inducing the production of anti-apoptotic factors BCL-2 (Mele et al., 2004), survivin (Liu et al., 2009), and osteoprotegerin (McCole et al., 2000; Castellanos-Gonzalez et al., 2008). Later in infection, in a different part of the *C. parvum* life cycle known as the sporozoite and merozoite life stages, inhibition is removed and apoptosis of the host cell is promoted (Mele et al., 2004; Liu et al., 2009).

### Interferons (IFNs)

**IFNs** are an essential component to the host response to *C. parvum*. The importance of **IFN-γ** is shown by an increased susceptibility to *C. parvum* infection in IFN-γ^-/-^ mice (Mead and You, 1998; Lacroix-Lamande et al., 2002) and wild-type neonate mice treated with anti-IFN-γ-antibodies (McDonald et al., 2013). Adult mice with a disrupted IFN-γ gene shed more parasites, experience extensive damage to the intestinal mucosa, and die within weeks of infection (Theodos et al., 1997). Severe combined immunodeficiency (SCID) mice, which are deficient in T and B cells, experience reduced *C. parvum* infection compared to SCID IFN-γ^-/-^ mice, showing that protective IFN-γ during *C. parvum* infection is derived, at least in part, from non-T or B cells (Hayward et al., 2000). In addition to increased IFN-γ, *in vivo* piglet infection and *in vitro* experiments show that intestinal epithelial cells secrete abundant **IFN-λ3** (a type-III IFN) independently of specialized immune cells (Ferguson et al., 2019). Historically, type III IFNs have been associated with local epithelial defense from viruses (Zhou et al., 2018). More recently, IFN-λ was shown to mediate the gut epithelium defense against non-viral pathogens via TLRs (Odendall et al., 2017). Neutralization of IFN-λ3 leads to increased villus blunting and fecal shedding of infective *C. parvum* in neonate mice, and when intestinal epithelial cells are primed with recombinant IFN-λ3 they show reduced barrier disruption and increased cellular defense against *C. parvum* (Ferguson et al., 2019).

### Specialized Immune Cells

#### Natural Killer (NK) Cells

NK cells contribute to the innate immune response to *C. parvum* through IFN-γ production and cytolysis of infected epithelial cells. *In vivo*, treating immunocompetent or immunodeficient mice with the NK cell activator, IL-12, leads to a protective effect against *C. parvum* associated with a concomitant rise in intestinal IFN-γ (Urban et al., 1996); *in vitro*, human NK cells lyse infected intestinal epithelial cells in response to IL-15 and presentation of MHC class I-related protein A and B (Dann et al., 2005). Mice lacking NK cells experience increased severity of infection and excrete more oocysts compared to mice with NK cells, but when treated with anti-IFN-γ antibodies the infection of NK positive mice was heavily exacerbated, thus implying a protective role of NK cells that is connected to IFN-γ (Barakat et al., 2009). Despite the increased morbidity in mice without NK cells, they produced IFN-γ after infection, meaning that NK cells are one, but not the only source of IFN-γ in response to *C. parvum*. The number of NK cells localized in the gut is increased within days following *C. parvum* exposure in lambs (Olsen et al., 2015). Activation of the NK cell receptor, NKG2D, is involved in NK cell-mediated protection, via its ligand, MICA, which is upregulated in the intestinal epithelium of infected humans (Dann et al., 2005). The role that other innate-like lymphocytes play during *C. parvum* infection is poorly understood and future investigations are warranted.

#### Dendritic Cells (DCs)

DCs exposed to *C. parvum* secrete numerous cytokines including IL-6, IL-1β, IL-12, IL-18, TNFα, and type I interferons via TLR4 receptor activation (Barakat et al., 2009; Bedi and Mead, 2012; Perez-Cordon et al., 2014). DCs also capture *C. parvum* antigens in the gut mucosa and migrate to draining lymph nodes where they present these antigens and facilitate the adaptive immune response (Auray et al., 2007; Perez-Cordon et al., 2014). DCs may acquire such antigens by directly capturing luminal organisms or phagocytizing apoptotic infected epithelial cells (Farache et al., 2013). Macrophages may further engulf free *C. parvum* and transfer the parasite to DCs for migration (Marcial and Madara, 1986). One hypothesis for the increased infection susceptibility of neonatal mice compared to adults is that neonates have fewer intestinal DCs, and injecting neonates with Flt3L – which induces DC differentiation from progenitor cells – increases the number of DCs as well as resistance to infection (Lantier et al., 2013). Furthermore, adult mice devoid of DCs are more susceptible to infection and excrete more parasites, and adoptive transfer of DCs pre-exposed to *C. parvum* reduces the parasite load (Bedi et al., 2014).

#### Macrophages

Macrophages develop from the same bone marrow precursor cells as DCs and are found in most organ systems and epithelial barriers, including the gut (Verschoor et al., 2012; Kumar, 2019). Following *C. parvum* infection in neonatal mice, macrophages accumulate in the lamina propria (de Sablet et al., 2016) and are associated with intact and digested parasites in Payer’s patches in guinea pigs (Marcial and Madara, 1986). Macrophages’ contribution to *C. parvum* clearance appears to be primarily as a secondary source of IFN-γ. Infected Rag2^-/-^ $\gamma_{c}^{-/-}$ mice, which lack T and B lymphocytes and NK cells, still produce IFN-γ, suggesting an IFN-γ source alternative to T cells and NK cells (Barakat et al., 2009). When treated with clodronate-liposomes to deplete macrophages, the mice were less resistant to *C. parvum* and could not produce IFN-γ (Barakat et al., 2009). IFN-γ production by macrophages is promoted by IL-18 when Rag2^-/-^ $\gamma_{c}^{-/-}$ mice are infected by *C. parvum* (Choudhry et al., 2012), and IFN-γ^-/-^ mice have fewer macrophages and T cells recruited to the gut accompanying an inability to recover from infection (Lacroix-Lamande et al., 2002).

#### Neutrophils

Neutrophils infiltrate the intestinal mucosa during *C. parvum* infection (Goodgame et al., 1995), and preventing mucosal recruitment of neutrophils increases *C. parvum*-related barrier dysfunction as measured by transepithelial electrical resistance (Zadrozny et al., 2006). Inhibiting neutrophil recruitment does not influence mortality or infection severity, nor does it affect *C. parvum*-mediated villous atrophy and diarrhea (Zadrozny et al., 2006). With no influence on mortality or infection severity, it does not appear that neutrophils are directly protective in the context of *C. parvum.*

**Figure 1:**
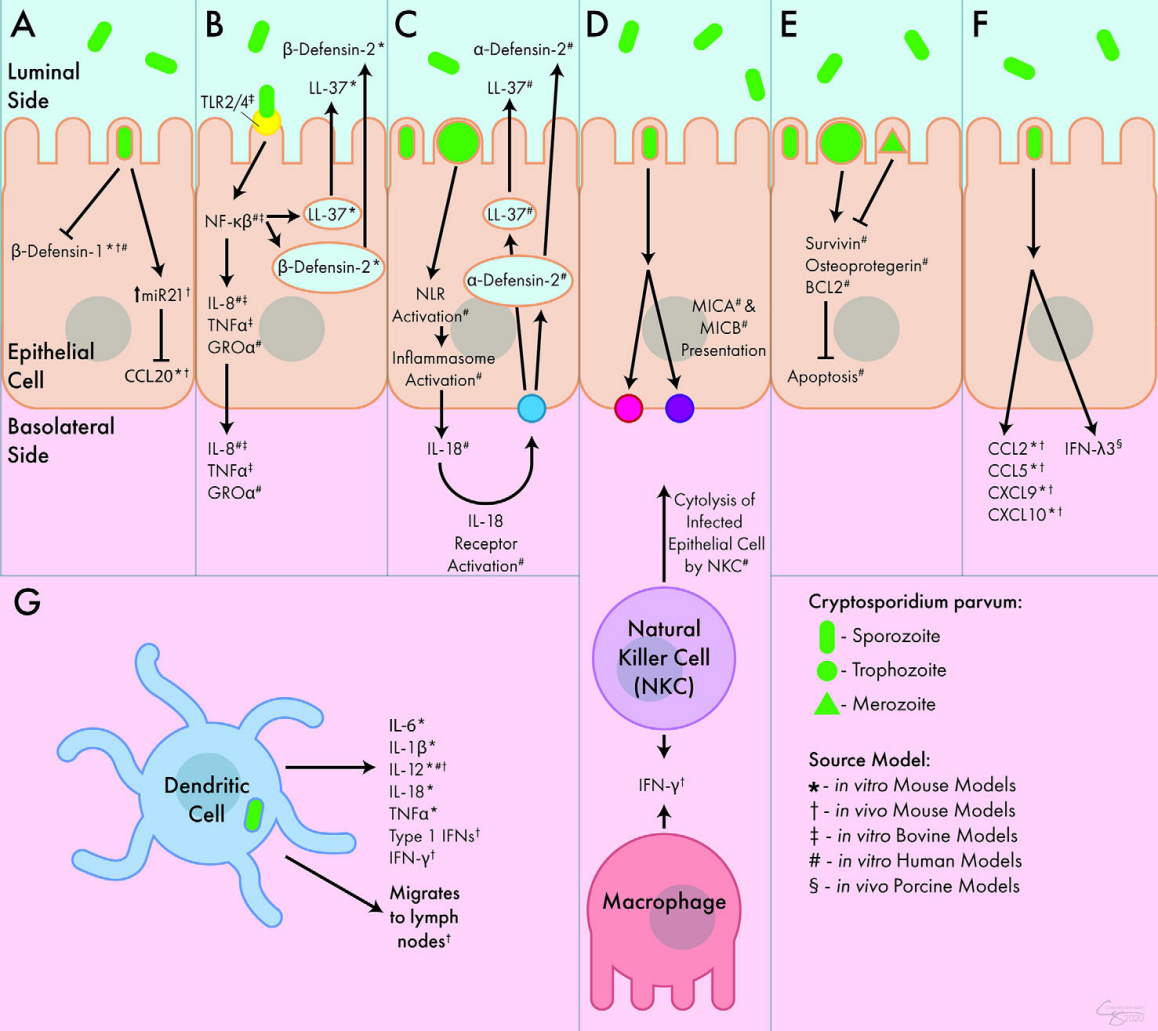
Innate Immune Response to *C. parvum*. **(A)** *C. parvum* inhibits the release of the antimicrobial peptides β-defensin-1 and CCL20. **(B)** Activation of TLR receptors by *C. parvum* leads to the luminal secretion of antimicrobial peptides β-defensin-2 and LL-37 as well as the basolateral secretion of IL-8, TNFα, and GROα. **(C)** Inflammasome activation by *C. parvum* leads to the basolateral release of IL-18, which causes the luminal secretion of α -defensin-2 and LL-37. **(D)** *C. parvum*-mediated presentation of MICA and MICB lead to cytolysis of infected epithelial cells by NK cells. NK cells and macrophages both act as sources of IFN-γ during infection. **(E)** *C. parvum* trophozoites stimulate apoptosis, but merozoites inhibit apoptosis, mediated through survivin, osteoprotegerin, and BCL2. **(F)** In response to *C. parvum*, intestinal epithelial cells release numerous chemokines and cytokines including CCL2, CCL5, CXCL9, CXCL10, and IFN-λ3. **(G)** DCs respond to *C. parvum* by releasing IL-6, IL-1β, IL-12, IL-18, TNFα, and type I interferons. They can also migrate to lymph nodes following parasite exposure. Interferon (IFN), Interleukin (IL-), Tumor Necrosis Factor (TNF), C-C Chemokine Ligand (CCL), C-X-C Chemokine Ligand (CXCL), Growth Regulated Oncogene (GRO), Toll-Like Receptor (TLR), Nod-Like Receptor (NLR), MicroRNA 21 (miR21), Nuclear Factor (NF), Cathelicidin (LL-37), Major Histocompatibility Complex Class I Chain-Related Protein (MIC), B-Cell Lymphoma 2-Apoptosis Regulator (BCL2), Natural Killer Cell (NKC).

As research models advance, the multi-dimensional innate immune response grows more complex but better understood (**Figure 1**). However, questions regarding the relevancy of these data to the natural hosts of *C. parvum* remain, provided the use of models that do not fully recapitulate the environment of human or ruminant intestines. New biotechnological advances, such as the development of bovine and human organoids, may provide the models necessary to confirm what is currently inferred about the innate immune response to *C. parvum* in these hosts.

## *Cryptosporidium* Research Models

The potential to fully understand *C. parvum*’s pathogenesis and develop therapeutics is dependent on the models used to research the host-pathogen interactions it induces within its natural and clinically relevant hosts (i.e. human and cattle). Traditional *in vitro C. parvum* infection models can only be maintained for several days at a time and do not fully recapitulate native intestinal tissue, and *In vivo* mouse models are sub-optimal, as mice are not a natural host of *C. parvum*. Innovative models and advancing technologies are necessary to advance this field.

### *In Vivo* Models

*In vivo* animal models are foundational to host-pathogen interaction research, but the nature of *C. parvum* complicates the application of traditional animal models. The natural and clinically relevant hosts for *C. parvum* are humans and ruminants; mice can sustain *C. parvum* infection but only when severely immunocompromised (Mead et al., 1991; Petry et al., 1995; Griffiths et al., 1998). Given that humans and ruminants are the primary natural hosts, calves, lambs, and non-human primates have been used to investigate cryptosporidiosis in naturally infected species (Tzipori, 1998). However, housing and maintaining large animal species requires significant funds and specialized facilities, equipment, and training. Moreover, many of the genetic and molecular research tools that are available for mice models are not available for large animal models such as cows or sheep.

Adult mice, a preferred animal model in terms of costs and availably of reagents, are resistant to *C. parvum* but are susceptible to infection by the related species, *C. muris*; however, *C. muris* differs from *C. parvum* in phylogeny, biochemical nature of infection, and infection site (*C. muris* infects the stomach mucosa) (Sateriale et al., 2019). Mice can become susceptible to *C. parvum* through chemical or genetic immunosuppression, such as the previously discussed SCID (Mead et al., 1991), IFN-γ^-/-^ (Griffiths et al., 1998), and Rag2^-/-^ mice, which, in addition to neonate mice, have provided established murine platforms for *C. parvum* research (Petry et al., 1995). Unfortunately, mouse models have limited translatability for natural hosts such as humans and cattle. This has been elucidated through bovine-specific responses to *C. parvum* that are absent in mice, such as differences in NK cell receptor activation (Allan et al., 2015), recruitment of γδ T cells (Guzman et al., 2012), and developed resistance in adulthood (Sateriale et al., 2019). More recently, *C. tyzzeri* was identified as a natural mouse pathogen that mirrors aspects of *C. parvum’s* pathogenesis and host response in mice (Sateriale et al., 2019).

### *In Vitro* Models

The allure of primary intestinal epithelium cells lies in the morphological and species-specific accuracy compared to immortalized cell lines. Primary human (Castellanos-Gonzalez et al., 2013) and bovine intestinal epithelial cells have been successfully infected with *C. parvum* (Hashim et al., 2006). Unfortunately, primary intestinal cells have limitations involving their availability, obsoletion, and difficulty in long-term propagation (Varughese et al., 2014).

Most *in vitro* models for *C. parvum* host-pathogen interaction research include cancer-derived transformed or immortalized human cell lines including HCT-8, Caco-2, and HT29 cells, which are all derived from colorectal adenocarcinomas (Karanis and Aldeyarbi, 2011). Other non-colorectal cancer cell lines have also been used: RL95-2 (human endometrial carcinoma) (Rasmussen et al., 1993), Madin-Darby bovine kidney cells (Upton et al., 1994), MRC-5 (lung fibroblast) (Dawson et al., 2004), FHs 74 Int cells (non-cancer, immortalized human small intestinal epithelium) (Varughese et al., 2014), and BS-C-1 (African green monkey kidney) cells (Deng and Cliver, 1998). None of these lines maintained infection longer than six days except for HT29 cells, which could maintain infection for thirteen days but only for the asexual life stages of *C. parvum*. One non-intestinal cell line, COLO-680N, is human esophageal squamous carcinoma-derived and can propagate infective parasites continually for eight weeks, but applications to host-pathogen interaction are questionable given that the esophagus is not the natural niche for *C. parvum* (Miller et al., 2018).

Early attempts to utilize three-dimensional structures for *C. parvum* research involved low-shear microgravity cultures where HCT-8 cells seeded onto submucosa grafts formed structures that maintained *C. parvum* infection; however, parasites decreased after 48 hours (Alcantara Warren et al., 2008). Later, a hollow fiber bioreactor system was used to infect three-dimensional HCT-8 cell structures for over six months, which is far longer than two-dimensional HCT-8 infection, while producing significantly more oocysts/day/mL (Morada et al., 2016). Silk fiber scaffolding has also been utilized to induce three-dimensional culture of Caco-2 and HT29 cells, maintaining infection for two weeks (DeCicco RePass et al., 2017).

While these cell lines are useful tools, they are susceptible to genetic variation, most cannot maintain all phases of the *C. parvum* life cycle, and most cannot maintain and propagate *C. parvum* infection for extended periods of time (Bhalchandra et al., 2018). This, in addition to the fact that these cell lines do not recapitulate the native intestinal epithelial tissue of *C. parvum* hosts, encourages the search for increasingly accurate models of study.

#### Enteroids

**Figure 2:**
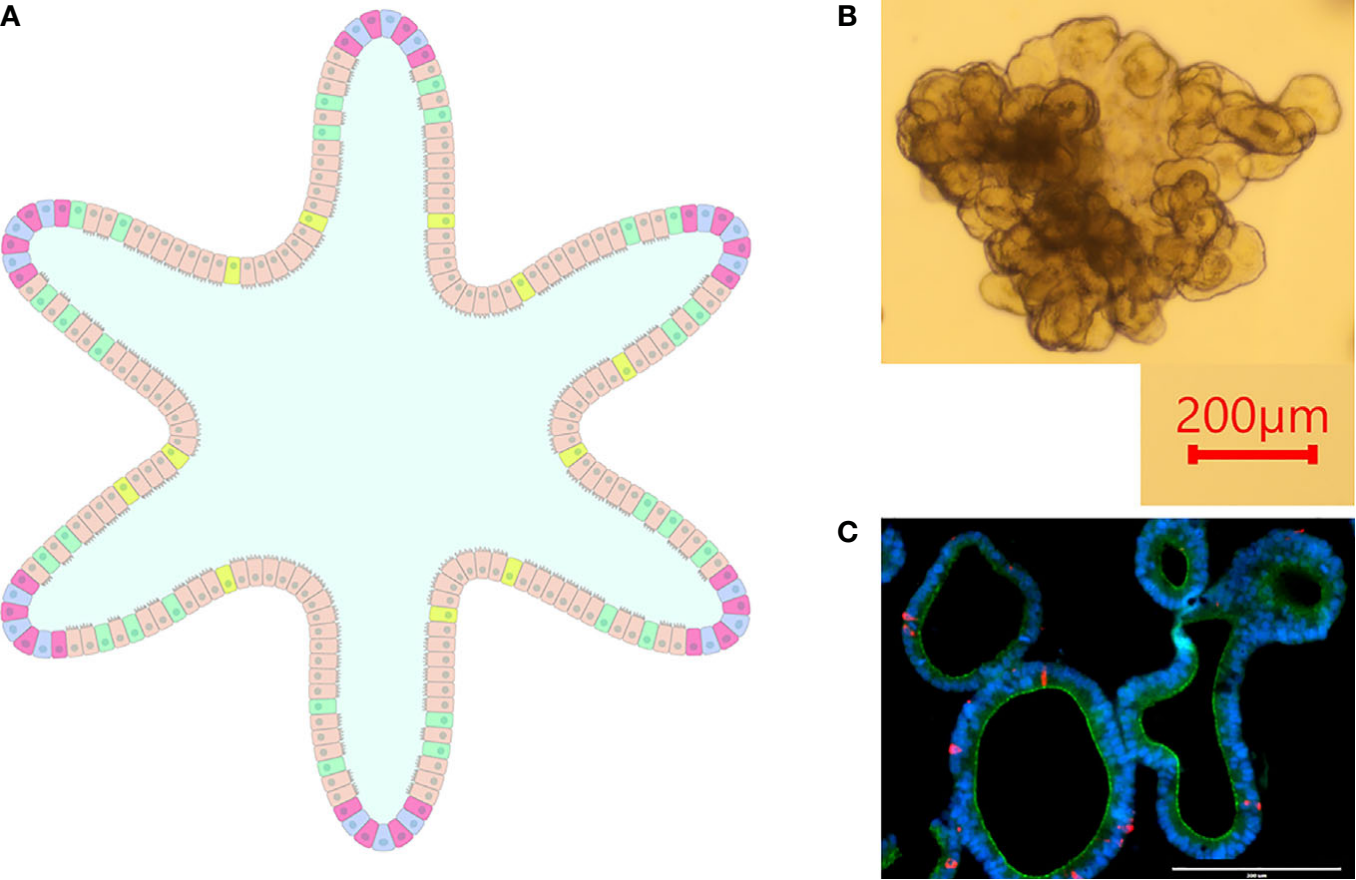
Intestinal Organoids. **(A)** graphical representation of an intestinal organoid. The inside of the organoid corresponds to the luminal side, and the outside of the organoid corresponds to the basolateral side. Blue: intestinal stem cells, Red: Paneth cells, Tan: enterocytes, Green: goblet cells, Yellow: enteroendocrine cells. **(B)** Bovine intestinal organoid 6 days post-plating. Numerous folds and budding structures are noted, indicating crypt and villi-like domains. **(C)** Sectioned ovine intestinal organoid illustrating nuclei (DAPI), apical junctional protein ZO-1 (green), and chromogranin-A (red) indicating enteroendocrine cell differentiation.

Intestinal organoids (aka enteroids) circumvent shortcomings exhibited by cell lines and primary epithelial cells while also introducing a three-dimensional culture model. Enteroids are composed of a polarized single layer of epithelium with crypt and villus domains containing the various intestinal epithelial cells such as stem cells, enterocytes, enteroendocrine cells, goblet cells, etc., thus recapitulating the microanatomy and functionality of native intestinal epithelial tissue (**Figure 2**) (Zachos et al., 2016).

Stem cell-derived organoids allow long-term three-dimensional culture while maintaining the morphological relevance of native tissue. Isolated crypts from neonatal and immunocompromised mice were exposed to *C. parvum* upon plating, resulting in inhibited organoid propagation and budding, decreased expression of intestinal stem cell markers, and increased cell senescence (Zhang et al., 2016). In another study, human enteroids were infected with *C. parvum* by microinjection and the parasite was able to complete its entire life cycle within these organoids (Heo et al., 2018). Though bovine enteroids have been described, they have not yet been used to study *C. parvum* infection (Powell and Behnke, 2017; Hamilton et al., 2018; Derricott et al., 2019).

Organoid technology for *C. parvum* research is in its relative infancy, but the benefits of the culture model are enticing and allow questions that were not possible to investigate with previous models.

## Discussion

*C. parvum* is a parasite of international clinical importance across human and animal healthcare. Because of its high infectivity, resistance to water treatment, and the danger it poses to immunocompromised individuals, understanding the responses it induces in its host is a high priority endeavor to allow the creation of effective preventative measures and therapies. The innate immune response is multifaceted and involves the intestinal epithelium, innate immune cells, and a complex interplay of cytokine signaling. To discover this, various models have been utilized. These include natural host species such as calves as well as more specialized models like immunocompromised mice, and *in vitro* models such as primary cell explants and immortalized cell lines. A relatively recent shift to three-dimensional cultures and the expanding use of organoids opens new avenues to study the parasite and its host-pathogen interaction.

As research in the field continues, attention must be brought to handling *C. parvum* from a ‘One Health’ perspective. New models must increase the relevant understanding of the parasite in bovine and human hosts and drive the discovery of innate mechanisms of resistance that can be utilized for management. Improved knowledge of the innate defenses against *C. parvum* in both ruminant and human hosts will hopefully lead to treatments to augment host natural innate defense and act as transient preventative measures to reduce environmental transmission of *C. parvum* between and within host species.

## Author Contributions

CC performed writing – original draft preparation. CC and AK performed writing – review and editing. AK performed project administration and supervision. All authors contributed to the article and approved the submitted version.

## Funding

The work presented here was supported by funding from The Center for Food and Animal Health of The National Institute of Food and Agriculture, grant number: CALV-RIVAS-0060.

## Conflict of Interest

The authors declare that the research was conducted in the absence of any commercial or financial relationships that could be construed as a potential conflict of interest.
